# Amyloidoma, an Unusual Cause of Fracture

**DOI:** 10.1155/2014/424056

**Published:** 2014-03-16

**Authors:** Frank Verhoeven, Clément Prati, Daniel Wendling

**Affiliations:** Rhumatologie, CHU Jean Minjoz, 2 Boulevard Fleming, 25030 Besançon, France

## Abstract

We report a case of a spontaneous hip fracture in a context of dysglobulinemia. The bone histologic examination found amyloidoma. Amyloidoma is an overload pathology and an unusual cause of fracture. In most of the cases, it is associated with myeloma and the difference between bone invasion of myeloma and amyloidoma in an osteolytic radiographic picture is not easy but is of importance because prognosis and treatment may be totally different. Thus, in the context of dysglobulinemia, one must keep in mind that spontaneous bone fracture may be due to amyloidoma with another prognosis.

## 1. Introduction

Amyloidoma is an uncommon cause of bone fragility. It occurs in the context of dysglobulinemia and the difference between amyloidoma and myeloma is difficult but is important.

## 2. Case

**Figure 1:**
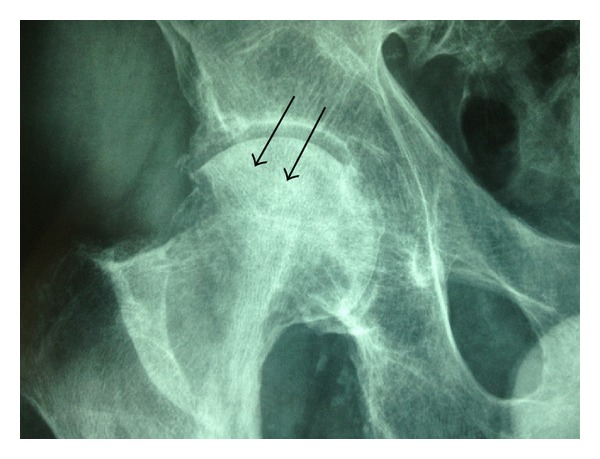
Radiographic aspect of hip fracture Garden 3 secondary to amyloidoma.

**Figure 2:**
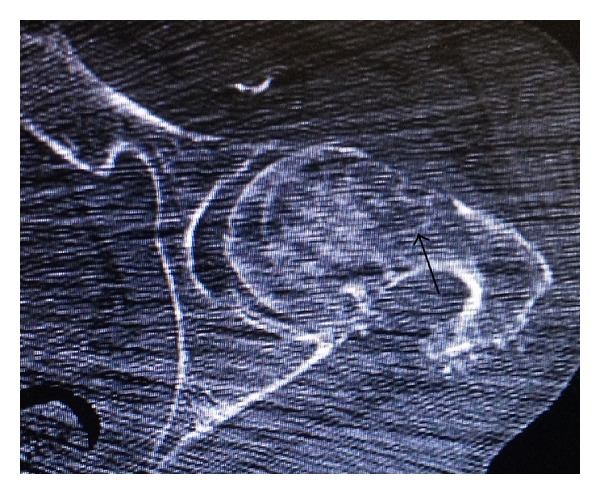
Geode of the left hip probably secondary to amyloidoma.

We report the clinical story of an 87-year-old man who was hospitalized for a right hip fracture without high energy trauma. He was known, but not followed up, for a kappa light chain monoclonal gammopathy diagnosed 7 years ago. He had no inflammatory syndrome (CRP < 10 mg/dL and ESR < 25 mm) and no hypercalcemia (<2, 57 mmol/L). The electrophoresis was characterized by an hypogammaglobulinemia and the presence of kappa light chain. The myelogram showed 9% of kappa light chain monoclonal plasmocytes. The Bence Jones proteinuria was positive. He underwent a hip arthroplasty and the histological examination of the femoral head found multifocal non-AA amyloid deposits, with osteolysis and a pseudotumoral aspect (amyloidoma). A biopsy of accessory salivary glands found non-AA amyloid deposits too. The radiographic exam (Figure 1) at the time of the fracture diagnosis showed geodes surrounded by area of osteosclerosis corresponding to focal deposits of amyloid. The extension statement showed only other geodes in the left hip (Figure 2). Six months later, he suffered from a contralateral hip fracture probably secondary to amyloidosis too.

## 3. Discussion

This is the second observation of hip fracture secondary to amyloidoma [1]. Amyloidoma is an unusual cause of fracture. Amyloidosis is an overload pathology characterized by extra cellular deposits of amyloid substance. The osteoarticular involvement represented by amyloidoma, amyloid arthropathy [2], and amyloid polyarthritis [3] is rare. Few types of amyloidosis are responsible for these manifestations [4]: AL amyloidosis which is primary or associated with myeloma [5], *β* 2-microglobulin amyloidosis which is associated with chronic dialysis [6], and ATTR amyloidosis including the senile and the hereditary amyloidosis.

Amyloidoma is most frequently associated with the AL amyloidosis which is linked with myeloma by the kappa or lambda light chain. These light chains share the same amino acid sequence with the AL amyloid substance [4]. Among these associations, amyloidoma can be a primary pathology, but it is less frequent. In the literature, 35 cases of bone locations (Table 1) are described to date and mainly in the spine. There are 23 vertebral involvements, and 6 are located at the skull. Peripheral involvements are more exceptional. There are just two humeral locations and one femoral [1] location described and finally 3 locations in the belts. This distribution is the same as for myeloma, in the trabecular bone. These locations might be explained by the possible immunoglobulin origin of amyloid proteins and the similarity of the amino acid sequence. The difference between bone invasion of myeloma and amyloidoma in front of an osteolytic lesion is not clear in a patient suffering from myeloma. In case of amyloidoma, we can observe a geode surrounded by osteosclerosis and this osteosclerosis may differentiate between the two conditions. This detail may be interesting in the absence of histologic data. This is important since the treatment and the prognosis are totally different between myeloma lesions and amyloidoma.

Thus, in case of fracture in a patient with dysglobulinemia, one must keep in mind the possibility of an amyloidoma, especially in front of a geode surrounded by osteosclerosis.

## Figures and Tables

**Table 1:** Bone locations of amyloidoma.

Authors	Locations	Context
Factor RE et al.; Diagn Cytopathol. (2012)	Iliac bone	Myeloma
Farrell K et al.; J Clin Oncol. (2011)	Cervical spine	Myeloma
Parmar H et al.; AJNR Am J Neuroradiol. (2010)	1 skull 1 lumbar spine 1 thoracic spine 1 iliac bone	Primary
Oruckaptan H et al.; Turk Neurosurg. (2009)	Skull and cervical spine	Dialysis
Abbas N et al.; Br J Neurosurg. (2008)	Thoracic spine	Primary
Iplikcioglu AC et al.; Spine. (2007)	Cervical spine	Primary
Volkan Aydin M et al.; J Spinal Disord Tech. (2006)	Thoracic spine	Primary
Iguchi T et al.; Rinsho Ketsueki. (2005)	Humerus	Dysglobulinemia
Mulleman D et al.; Eur Spine J. (2004)	Cervical spine	Primary (evolution to systemic amyloidosis)
Manucha V et al.; Diagn Cytopathol. (2003)	Thoracic spine	Primary
Rachbauer F et al.; AJR Am J Roentgenol. (2003)	Clavicle	Dysglobulinemia
Unal A et al.; Clin Neurol Neurosurg. (2003)	Thoracic spine	Primary
Bruninx G et al.; J Radiol. (2001)	Sacrum	Primary
Hsu CW et al.; Ren Fail. (2001)	Cervical spine	Dialysis
Simoens WA et al.; AJNR Am J Neuroradiol. (2000)	Skull	Primary
Hwang SS et al.; AJNR Am J Neuroradiol. (2000)	Cervical spine	Primary
Miossec P et al.; Diabetes Metab. (1999)	Lumbar spine	Primary
Sancho JM et al.; Med Clin (Barc). (1999)	Lumbar spine	Primary
Mathew JM et al.; Br J Neurosurg. (1998)	Thoracic spine	Primary
Dee CH et al.; Spine. (1998)	Thoracic spine	Primary
Porchet F et al.; Spine. (1998)	Cervical spine	Primary
Pambuccian SE et al.; Am J Surg Pathol. (1997)	1 cervical spine 1 scapula 1 humeral head	Dysglobulinemia and lymphoplasmacytic infiltrate
Hidalgo F et al.; Neuroradiology. (1996)	Skull	—
Cloft HJ et al.; AJNR Am J Neuroradiol. (1995)	Thoracic spine	Primary
Chang YS et al.; Zhonghua Yi Xue Za Zhi (Taipei). (1993)	Thoracic and lumbar spine	Primary
Unal F et al.; J Neurosurg. (1992)	Skull	Primary
Dickman CA et al.; Neurosurgery. (1988)	Cervical spine	—
Leeson MC et al.; Spine. (1985)	Thoracic spine	Primary
Lai KN et al.; Am J Med. (1984)	Femoral neck	Myeloma
Giordano A et al.; Otolaryngol Head Neck Surg. (1983)	Skull	—
FADELL EJ et al.; Am J Surg. (1964)	Sternum	—

## References

[1] Lai KN, Chan KW, Siu DL (1984). Pathologic hip fractures secondary to amyloidoma. Case report and review of the literature. *American Journal of Medicine*.

[2] Fautrel B, Fermand J-P, Sibilia J, Nochy D, Rousselin B, Ravaud P (2002). Amyloid arthropathy in the course of multiple myeloma. *Journal of Rheumatology*.

[3] Prokaeva T, Spencer B, Kaut M (2007). Soft tissue, joint, and bone manifestations of AL amyloidosis: clinical presentation, molecular features, and survival. *Arthritis and Rheumatism*.

[4] M'bappé P, Grateau G (2012). Osteo-articular manifestations of amyloidosis. *Best practice & research: Clinical rheumatology*.

[5] Sipe JD, Cohen AS (2000). Review: history of the amyloid fibril. *Journal of Structural Biology*.

[6] Sipe JD, Benson MD, Buxbaum JN (2012). Amyloid fibril protein nomenclature: 2012 recommendations from the Nomenclature Committee of the International Society of Amyloidosis. *Amyloid*.

